# High exposure to malaria vector bites despite high use of bednets in a setting of seasonal malaria in southwestern Mali: the urgent need for outdoor vector control strategies

**DOI:** 10.1186/s13071-025-06818-8

**Published:** 2025-07-09

**Authors:** Sophie Sarrassat, Mahamoudou Toure, Mohamed Traore, Ayouba Diarra, Hamady Coulibaly, Abdoul Zamilou Arou, Cheick Oumar Tangara, Gunter Muller, John Carlton Beier, John Vontas, John Bradley, Sekou F. Traore, Seydou Doumbia, Immo Kleinschmidt

**Affiliations:** 1 Department of Infectious Disease Epidemiology, London School of Hygiene and Tropical Medicine (LSHTM), Keppel Street, London, WC1E 7HT UK; 2 University Clinical Research Centre/University of Sciences, Techniques and Technologies of Bamako (UCRC/USTTB), Bamako, Mali; 3 University of Miami Miller School of Medicine, Miami, FL USA; 4 Institute of Molecular Biology & Biotechnology/Foundation for Research and Technology, Athens, Greece; 5 Agricultural University of Athens, Athens, Greece; 6 Wits Research Institute for Malaria, School of Pathology, Faculty of Health Sciences, University of the Witwatersrand, Johannesburg, South Africa

**Keywords:** Outdoor and indoor malaria vector bites, Residual malaria transmission, Malaria vector control, Mali

## Abstract

**Background:**

Early evening and outdoor biting by vector mosquitoes undermines the effectiveness of insecticide-treated nets (ITNs), as users of nets are exposed to vector biting whilst not under a net, both outdoors and indoors. This study assessed exposure to malaria vector bites amongst users and non-users of ITNs in southwestern Mali.

**Methods:**

Using cross-sectional household survey data of human behaviour and malaria infection prevalence, along with mosquito human landing catch (HLC) data collected in 30 separate communities, the average number of *Anopheles gambiae* sensu lato (s.l.) mosquito bites per person per night (bppn) received outdoors and indoors were estimated for each survey respondent. The proportion of bites that were not preventable by using a net, the relative contributions of outdoor and indoor residual biting, and the risk factors for exposure to vector bites were estimated.

**Results:**

Despite very high use of nets (93.2%), malaria infection prevalence was 34% overall. A large proportion of respondents (78%) reported being outdoors at 8 pm, but by midnight, 98% were indoors. Net users were exposed to indoor biting for 1 h, on average, between going indoors and going to bed. For 91%, the net used was an ITN. Human biting rates peaked between 2 and 4 am, when most people (90%) were in bed. Individuals using a net received 11.2 bppn in total, of which 7.1 bppn (63%) occurred outdoors. Those not using a net received almost 10 times the number of bites indoors as net users (38.4 bppn versus 4.0 bppn). The total number of bites received by net users was about one third the total number of bites received by non-net users, indicating the proportion of bites not preventable by use of a net alone. Risk factors for biting exposure included not using a net, going indoors late, location near the river and age over 15 years.

**Conclusions:**

ITNs substantially reduce exposure to indoor biting, but in this setting, net users still received a large number of *Anopheles* mosquito bites, giving rise to high malaria infection prevalence despite near-universal net use. Most residual biting occurred outdoors, but about a third still occurred with individuals indoors before going under a net. Effective interventions that reduce residual outdoor and indoor biting are necessary to reduce the high malaria burden in settings like southwestern Mali.

**Graphical Abstract:**

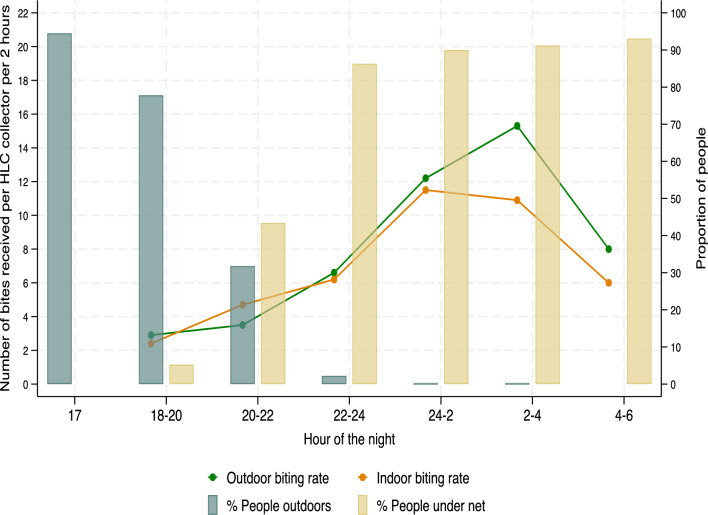

**Supplementary Information:**

The online version contains supplementary material available at 10.1186/s13071-025-06818-8.

## Background

Insecticide-treated nets (ITNs) have been the core protection against malaria for more than 20 years, with more than 3.1 billion nets delivered between 2004 and 2023 [1]. Their effectiveness is well established through randomised controlled trials [2], and modelling studies have shown that large-scale deployment of nets has been the main reason for the significant reductions in malaria cases and deaths over the period 2000–2015 [3]. However, declining trends in malaria burden have stalled over recent years, with an estimated global total of 597,000 deaths and 263 million malaria cases in 2023 [1]. In some countries, there has been no progress towards reducing the malaria burden despite the widespread roll-out of this intervention.

Vector dynamics (mosquito biting and resting behaviours) and insecticide resistance have been identified as important factors compromising the effectiveness of nets [4]. Residual malaria transmission has been defined [5, 6] as the transmission that continues despite optimal deployment of standard vector control interventions such as ITNs or indoor residual spraying (IRS). Early evening and outdoor biting by vector mosquitoes have been reported by a number of studies, often associated with changes in species composition resulting from widespread use of insecticides [7, 8]. The protective efficacy of ITNs relies on preventing biting by *Anopheles* mosquitoes when people are under a net and by killing mosquitoes touching or landing on the net. They do not provide personal protection against mosquito bites when people are outdoors or indoors but not using them.

In Mali, reported malaria deaths have gradually declined to 14,203 in 2023, whilst annual malaria cases have increased since 2000 to over 8 million in 2023 [1], despite the widespread implementation of World Health Organization (WHO)-recommended interventions, including ITNs as well as complementary interventions such as seasonal malaria chemoprevention (SMC) for children under 5 years of age (hereafter “children under 5”) and intermittent preventive treatment in pregnancy (IPTp). The purpose of this study was to assess exposure to malaria vector bites, outdoors and indoors, amongst users and non-users of ITNs in southwestern Mali, using cross-sectional household survey data on human behaviour and malaria infection prevalence, and concomitant entomological data on mosquito human landing catches (HLC) collected in random samples of the same communities. Human and entomological data were combined to estimate the average number of *Anopheles gambiae* sensu lato (s.l.) mosquito bites received per person per night, the proportion of bites that were not preventable by using a net, risk factors for biting exposure and the relative contribution of outdoor versus indoor biting, since this has implications for the suitability of supplementary vector control tools. The study was conducted during the baseline year of a cluster-randomised controlled trial (CRCT) evaluating the public health efficacy of attractive targeted sugar bait (ATSB) stations against malaria [9].

## Methods

### Study site

The study was conducted in the Koulikoro region, approximately 60 km southeast of Bamako in southwestern Mali. In this region, malaria is seasonal, with high numbers of malaria cases occurring between July and December each year. The primary malaria vector is *Anopheles coluzzii*, with *An. gambiae* sensu stricto (s.s.) and *An. arabiensis* also contributing towards transmission [10]. Pyrethroid resistance has been reported in *An. gambiae* s.l. [11, 12]. According to the 2021 Malaria Indicator Survey (MIS) [13], 95% of households in this region owned at least one ITN, 75% owned at least one ITN per two persons (universal coverage), 72% of respondents reported having slept under an ITN the night prior to the interview, and 42% of women who had a live birth in the past 2 years had received three doses or more of sulfadoxine/pyrimethamine for prevention of malaria in pregnancy. Amongst children under 5, 23% tested positive by rapid diagnostic test (RDT).

### Human behaviour and malaria infection cross-sectional data

A cross-sectional household survey was conducted during the peak of the malaria season, from 3rd to 15th November 2021, in 80 communities which formed the trial clusters for the subsequent CRCT on ATSB stations [9]. This survey assessed, prior to ATSB deployment, the community prevalence of malaria infection and people’s behaviours related to exposure to mosquito bites.

Using lists from a population census performed in the study area in March 2021, individuals aged 6 months or older were randomly selected in each community to be tested for malaria parasites using RDTs (Bioline™ Malaria Ag P.f/Pan). The sample size was determined by the requirements of the subsequent CRCT [9].

A structured questionnaire adapted from the Roll Back Malaria (RBM) MIS [14] was administered to all members of households in which one or more individuals had been sampled for RDT testing. Following informed written consent, the household head was interviewed on housing characteristics and net ownership. All consenting household members were then interviewed about malaria preventive behaviours including time of going indoors, time of going to bed and net use the night prior to the interview. Caregivers responded on behalf of their children. Detailed procedures were published as part of the ATSB CRCT protocol [9].

### Entomological monthly data

Thirty of the 80 communities were randomly selected for entomological monitoring, subject to inclusion criteria of accessibility and community consent. Each month, 15 households per community were randomly selected for entomological data collection from the census list, yielding on average 12 households per community after allowing for non-responses.

Using standard methods, HLC were performed one night per month at two of the selected households in each community [9]. Centers for Disease Control and Prevention (CDC) light trap collections, which were conducted at the remaining households, were not used for the present study since only HLC data provide a direct measure of human exposure to mosquito bites. At the HLC houses, collectors using an aspirator and a flashlight caught mosquitoes landing on their exposed legs, from 6 pm to 6 am in 2-hourly periods. One collector was positioned at a designated location inside the house and one outside. Collectors rotated capture positions (indoor/outdoor) every 2 h throughout their shift. Captured mosquitoes were stored in labelled test tubes according to the hour and location of capture, and specimens were morphologically identified to determine species.

### Data analysis

Data analyses were restricted to the 30 communities which had both entomological and household survey data, and to individuals who reported having slept at home the night prior to the interview. Statistical analyses accounted for within-community correlation of responses using robust standard errors, and were performed in Stata 18 software.

All *An. gambiae* s.l. females were included in the analysis, and their counts were used to estimate the human biting rate (HBR) for each 2-h interval from 6 pm to 6 am, indoors and outdoors. Community-level estimates of 2-hourly HBR for the months of September, October and November 2021 were averaged to produce one estimate of HBR per 2-h interval per community. The HBRs for each community were merged with the corresponding cross-sectional survey data collected in November 2021 for the same community. Two-hourly indoor and outdoor HBR were assumed to be the same for all respondents of a given community.

The number of malaria vector bites each respondent received during the night before the survey (hereafter referred to as the number of vector bites per person per night [bppn]) was estimated using respondents’ self-reported time of going indoors and under a net, if they did. Individual exposure to outdoor biting was calculated as the cumulative outdoor HBR for the individual’s community, from 6 pm to the time the respondent reported going inside. For individuals who reported having gone indoors at 5 pm, the outdoor biting exposure was set as zero. For individuals who did not use a net, the indoor biting exposure was calculated as the cumulative indoor HBR from the time they went indoors until 6 am. Individuals who used a net were assumed to be not exposed to mosquito bites once they went to bed, and their indoor biting exposure was calculated as the cumulative indoor HBR from the time they went indoors to the time they went to bed and under a net. We did not have data on the time they got out bed and the time they went outdoors in the morning. The proportion of bites that cannot be averted by the use of a net alone was calculated as the average total bites (indoors and outdoors) received by net users divided by the average total bites received by non-net users.

Poisson regression models with robust standard errors to account for within-community correlation of responses were used to identify risk factors for the number of bppn (individual biting exposure) and to compute crude and adjusted rate ratios (RRs) of biting relative to reference categories of potential risk factors. Household wealth index was computed from the first component of a principal component analysis of household assets and goods reported by household heads [15]. Risk factors were selected for inclusion in the multivariable model if associated with exposure to mosquito biting, with *P* < 0.10.

Malaria infection prevalence was measured as the proportion of positive RDTs among individuals tested. Logistic regression with robust standard errors was used to compute crude and age-adjusted odds ratios (ORs) for the association between exposure to bites (number of bites received per person per night) and malaria infection.

## Results

A total of 6477 *An. gambiae* s.l. female mosquitoes were caught by HLC, most (69.2%) in September, and about 53% outdoors. In the household survey, 6211 respondents who resided in the households of those selected for RDT were interviewed, of whom 5751 (93%) reported having slept at home the night before the interview. Of these, 2997 (52%) were allocated an estimated individual biting exposure. For the remaining 2754 individuals, biting exposure could not be estimated because times of going indoors and/or under a net were missing. Distributions of basic socio-demographic characteristics (socio-economic status, age, sex, school attendance) and risk factors for malaria (household distance to the river, altitude and bednet use) were similar between individuals with missing data and those that were included in the analysis (Supplementary material, Table S1). Among the 749 tested by RDT, 672 slept at home the night before the interview and only 389 (59%) could be allocated an estimated individual biting exposure.

**Fig. 1:**
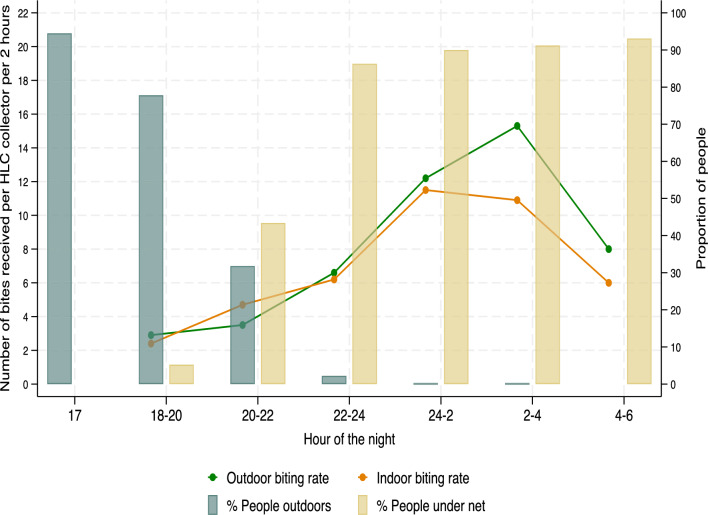
Outdoor and indoor human biting rate (average number of bites received per HLC collector per 2 h across clusters and from September to November 2021) and proportion of individuals outdoors and under a net by hour of the night

In the hours up to midnight, HLC collectors received between 2.9 and 6.6 outdoor bites per 2 h and between 2.4 and 6.2 indoor bites per 2 h on average across communities (Fig. 1). From midnight, the average HBRs increased, peaking at 15.3 bites per collector between 2 and 4 am for outdoor biting and at 11.5 bites per collector between midnight and 2 am for indoor biting. Between 4 and 6 am, HBRs were still eight and six outdoor and indoor bites per collector per 2 h. Across communities, the total bites per collector per night, from 6 pm to 6 am, ranged from 11.8 to 228.7, and the 2-hourly HBRs showed similar patterns across communities (Supplementary material, Figures S1–S3, Table S2).

As the proportion of people outdoors decreased through the evening and night, the proportion of people under a net increased (Fig. 1). At 8 pm, 78% of individuals reported being outdoors, at 10 pm 32% and by midnight 2%, whilst very few (0.1%) stayed outdoors up to 4 am. The proportion of respondents of all ages who reported net use the previous night was 93.2% (95% CI [88.2, 96.1]), and for 90.9% of net users the net used was an ITN (9.7% did not know the net brand) (Table 1). The majority of nets reported in the survey were pyrethroid only nets, but a small proportion (9%) were piperonyl butoxide (PBO) nets, and 9% were of unknown brand. Bednet use was similar across age groups. About an hour elapsed, on average, between the time a respondent went indoors and the time they went to bed.

**Table 1:** Bednet use (night prior to the interview) by age group

Age group	Bednet use (night prior to the interview)				Time (minutes) between going indoors and under a net (among net users)			
	Total	%	95% CI		Total	Mean	95% CI	
0–4 years	390	96.4	91.2	98.6	376	55.1	30.0	80.3
5–14 years	1157	91.9	85.6	95.6	1060	68.2	48.9	87.4
15–44 years	959	93.0	88.4	95.9	891	71.7	51.7	91.7
45+ years	490	93.9	87.6	97.1	459	64.2	42.7	86.8
All ages	2997	93.2	88.2	96.1	2787	66.9	48.1	85.6

**Fig. 2:**
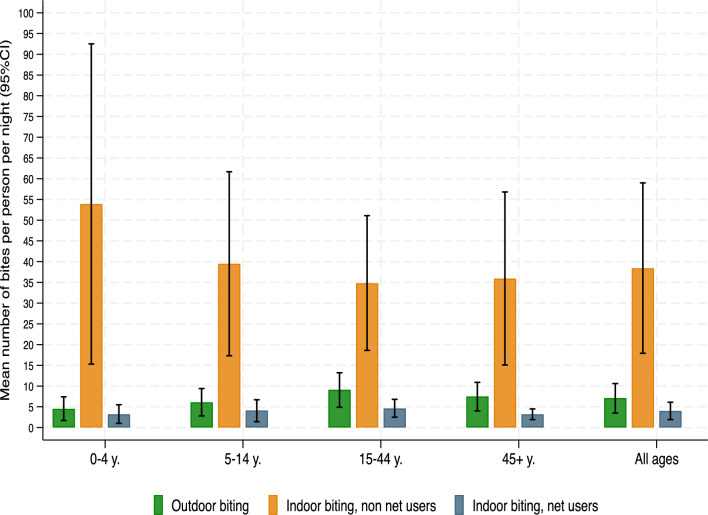
Individual outdoor and indoor biting exposure by age group (mean number of malaria vector bites received per person per night)

Respondents (users and non-users of nets) received an average of 7.1 bppn outdoors (Table 2 and Fig. 2). Indoor bites received were 4.0 bppn (95% CI [1.9, 6.1]) for net users and 38.4 bppn (95% CI [17.9, 59.0]) for those not using a net. Net users received on average a total of 11.2 bppn, of which 63% (7.1 bppn) were outdoors. The average total bites received per night varied with age: among net users, young adults (15–44 years old) received the highest average number of bites indoors (4.6 bppn) and outdoors (9.1 bppn). Children under 5 who used a net received the lowest average number of bites indoors (3.2 bppn) and outdoors (4.5 bppn). The very small subgroup of children under 5 who did not sleep under a net (*n* = 14) received the highest average number of bites indoors (53.9 bppn), although with large uncertainty around the point estimate (95% CI [15.3, 92.5]).

**Table 2:** Individual outdoor and indoor biting exposure by age group (mean number of malaria vector bites received per person per night)

Biting exposure	Age group	Bednet use	Total	Mean	95% CI	
Outdoor biting	0–4 years	–	390	4.5	1.7	7.4
	5–14 years	–	1157	6.1	2.8	9.4
	15–44 years	–	959	9.1	4.9	13.2
	45+ years	–	490	7.5	4.0	10.9
	All ages	–	2997	7.1	3.5	10.6
Indoor biting	0–4 years	Non-users	14	53.9	15.3	92.5
		Users	376	3.2	1.0	5.5
	5–14 years	Non-users	94	39.5	17.3	61.7
		Users	1063	4.1	1.4	6.7
	15–44 years	Non-users	67	34.8	18.6	51.1
		Users	892	4.6	2.5	6.8
	45+ years	Non-users	30	35.9	15.1	56.8
		Users	460	3.2	1.9	4.5
	All ages	Non-users	205	38.4	17.9	59.0
		Users	2792	4.0	1.9	6.1

Across all ages the proportion of bites that could not be averted by use of a net was estimated at 26.1% of total bites (average total bites received by net users divided by the average total bites received by non-users) (Table 3). This proportion was lower in children under 5, at 14.2%, and somewhat higher in adults aged 15–44 years, at 33.8%.

**Table 3:** Proportion of bites that cannot be averted by net use (average total bites received by net users divided by average total bites received by non-net users)

Age group	Bednet use	Total	Mean	95% CI		Ratio (%)
All ages	Users	2792	11.2	6.0	16.5	–
	Non-users	205	42.9	22.1	63.7	26.1
0–4 years	Users	376	7.9	3.5	12.3	–
	Non-users	14	55.5	17.7	93.2	14.2
5–14 years	Users	1063	10.3	4.8	15.8	–
	Non-users	94	43.2	21.0	65.5	23.8
15–44 years	Users	892	13.9	8.1	19.8	–
	Non-users	67	41.1	23.9	58.2	33.8
45+ years	Users	460	10.9	6.7	15.2	–
	Non-users	30	40.1	18.6	61.6	27.2

Factors associated with individual biting exposure are shown in Table 4. In the univariable Poisson regression analysis, net use the previous night, screen at the sleeping building windows, increased distance to the river and altitude were associated with reduced biting. In contrast, being aged 15–44 years old and going indoors later than 10 pm were associated with higher biting exposure. In the multivariable analysis, there was strong evidence that net use (RR = 0.26; 95% CI [0.16, 0.40]; *P* < 0.001) and distance ≥ 10 km versus within 5 km of the river (RR = 0.22; 95% CI [0.11, 0.44]; *P* < 0.001) were protective against biting. The proportion of people living in houses with window screens was very small (3%), and there was some evidence of a protective effect against biting exposure (RR = 0.72; 95% CI [0.56, 0.92]; *P* = 0.010). In contrast, there was strong evidence that age and later time of going indoors were risk factors for increased biting. Young adults, aged 15–44 years, were at highest risk compared to children under 5: RR = 1.25 (95% CI [1.05, 1.50]; *P* = 0.014). The later people were going indoors, the more they were exposed to biting: RR = 1.55 (95% CI [1.12, 2.14]; *P* = 0.008) comparing those going indoors after midnight with those going indoors before 10 pm.

**Table 4:** Risk factors for biting exposure (number of bites per person per night) (Poisson regression analysis)

	Total	Mean number of bites	Univariable analysis					Multivariable analysis (*N* = 2770)				
			IRR	95% CI		*P*-value		IRR	95% CI		*P*-value	
Household wealth quintiles												
Poorest	511	15.9	1.00	–	–	–	0.032	1.00	–	–	–	0.179
Poorer	516	10.9	0.68	0.45	1.05	0.081		0.90	0.65	1.25	0.538	
Middle	602	13.5	0.85	0.50	1.46	0.560		1.11	0.76	1.61	0.592	
Richer	657	14.8	0.93	0.53	1.63	0.798		1.11	0.80	1.53	0.541	
Richest	711	12.2	0.77	0.46	1.26	0.294		0.73	0.43	1.25	0.254	
Household distance to the river (km)												
< 5	279	32.7	1.00	–	–	–	< 0.001	1.00	–	–	–	< 0.001
[5–10]	932	20.2	0.62	0.33	1.16	0.136		0.56	0.29	1.10	0.095	
[10–60]	1786	6.8	0.21	0.11	0.40	< 0.001		0.22	0.11	0.44	< 0.001	
Household altitude (m)												
[320–380]	912	19.3	1.00	–	–	–	0.043	1.00	–	–	–	0.252
[380–400]	1386	12.4	0.64	0.39	1.06	0.085		0.81	0.61	1.06	0.129	
[400–510]	699	7.8	0.40	0.20	0.82	0.013		0.79	0.52	1.19	0.258	
Age group (years)												
0–4	390	9.6	1.00	–	–	–	< 0.001	1.00	–	–	–	0.005
5–14	1157	13.0	1.36	1.17	1.57	< 0.001		1.16	1.02	1.32	0.029	
15–44	959	15.8	1.64	1.35	2.01	< 0.001		1.25	1.05	1.50	0.014	
45–95	490	12.7	1.32	1.01	1.73	0.040		1.13	0.94	1.36	0.190	
Sex												
Male	1488	14.1	1.00	–	–	–	–					
Female	1509	12.7	0.90	0.77	1.06	0.198	–					
Time going indoors												
< 22 h	2043	11.6	1.00	–	–	–	< 0.001	1.00	–	–	–	0.006
[22–24 h]	887	16.7	1.44	1.21	1.72	< 0.001		1.30	1.10	1.54	0.003	
[24 h+]	67	23.4	2.01	1.56	2.60	< 0.001		1.55	1.12	2.14	0.008	
Bednet use prior night												
No	205	42.9	1.00	–	–	–	–	1.00	–	–	–	–
Yes	2792	11.2	0.26	0.14	0.48	< 0.001	–	0.26	0.16	0.40	< 0.001	–
Screen at the sleeping building's windows^a^												
Windows without screen	2659	14.2	1.00	–	–	–	–	1.00	–	–	–	–
All windows with screen	112	6.6	0.47	0.31	0.70	< 0.001	-	0.72	0.56	0.92	0.010	–

^a^Window characteristics collected for a maximum of three windows only

Malaria infection prevalence was 33.8% (95% CI [29.0, 38.9]) overall, varying substantially by age, with prevalence of 50% or more in children versus 20% or less in adults (Table 5). Prevalence was lower among net users, 33.1% (95% CI [28.3, 38.2]), compared to non-users, 42.3% (95% CI [28.4, 57.6]).

**Table 5:** Malaria infection prevalence, by age group and bednet use

Age group	Bednet use	Total	*P* (%)
All ages	All	672	33.8
	Users	620	33.1
	Non-users	52	42.3
0–4 years	All	69	49.3
	Users	67	50.8
	Non-users	2	–
5–14 years	All	210	58.1
	Users	194	57.7
	Non-users	16	62.5
15–44 years	All	269	20.5
	Users	247	18.2
	Non-users	22	45.5
45+ years	All	124	12.9
	Users	112	12.5
	Non-users	12	16.7

In the much smaller sample (*n* = 389) for whom data on infection status and biting exposure were available, the odds of malaria infection prevalence increased with increasing number of bites per person per night, but the statistical evidence for this association was very weak (Table 6).

**Table 6:** Association between biting exposure (number of bites per person per night) and malaria infection prevalence (logistic regression analysis)

	Total	Malaria infection prevalence (%)	Age-adjusted OR	95% CI		*P*-value	
Outdoor bites							
≤ 2	155	27.7	1.00	–	–	–	0.173
[2–4]	56	35.7	1.59	0.74	3.43	0.233	
[4–10]	77	40.3	2.80	1.25	6.31	0.013	
[10–20]	64	31.3	1.62	0.83	3.18	0.161	
> 20	55	30.9	1.82	0.91	3.64	0.088	
Total	407						
Indoor bites							
≤ 2	268	32.5	1.00	–	–	–	0.138
[2–4]	14	21.4	0.59	0.28	1.27	0.180	
[4–10]	45	35.6	1.26	0.58	2.75	0.555	
[10–20]	32	31.3	1.33	0.51	3.49	0.557	
> 20	30	43.3	1.97	0.76	5.11	0.161	
Total	389						
All bites							
≤ 2	87	31.0	1.00	–	–	–	0.223
[2–4]	52	38.5	1.68	0.68	4.10	0.258	
[4–10]	73	35.6	1.91	0.87	4.18	0.105	
[10–20]	88	26.1	1.31	0.63	2.75	0.470	
> 20	89	37.1	2.30	1.07	4.90	0.032	
Total	389						

## Discussion

Universal coverage of ITNs is recommended by WHO as a key intervention against malaria in countries with a high malaria burden [16]. In most countries, it has proved difficult to attain high coverage; in 2023, on average, 52% of the population at risk of malaria in the WHO African region were estimated to have slept under a net. The population in this study were therefore unusual in that nearly everyone (93%) reported having used a net, possibly as a result of effective distribution and behaviour change communication, or as a consequence of the high number of vector mosquito bites that residents are exposed to, as documented in the study. However, despite this very high use of nets, we found an overall prevalence of infection with malaria parasites of 34%. Clearly, high usage of nets may reduce malaria at a population level, but nets provide at best partial protection.

Nets were highly effective in reducing exposure to vector mosquito bites indoors, from 38.4 bppn for those not using a net to 4.0 bppn for those using a net, a reduction of 90% (Table 2). This large reduction was due in part to the late biting peak, which occurred when most people were in bed and under a net (Fig. 1). Users and non-users were on average exposed to 7.1 bppn outdoors, mostly during the early part of the night before going indoors. Thus, net users still received on average 11.2 bppn in total (Table 3), explaining the high malaria infection prevalence (33%) even amongst this group. The average total bites received per night varied with age, largely explained by different behaviours across age groups. Children under 5 spent the shortest time outside during the night and the longest hours under a net and therefore averted a large proportion of bites by using a net (86%), as opposed to young adults who went under a bednet later in the night and averted 66% of bites by using a net (Table 3). Children under 5 are therefore more efficient users of nets and should be prioritised for net use.

For net users, outdoor biting represents a large proportion (63%) of the residual bites they received. In this setting, therefore, additional indoor interventions would only be able to play a limited role in reducing overall exposure to vector mosquito bites, unless these interventions were highly effective in reducing mosquito populations. This demonstrates the need for interventions that reduce outdoor biting or overall biting by reducing the vector population. One such method would be ATSB stations, which were trialled in the study area but which did not demonstrate evidence of an effect large enough to detect [17]. Other potential future interventions would be releases of genetically modified mosquitoes or sterile insect techniques if these can be shown to be effective and practical for large-scale deployment.

In this study, people who did not use nets were estimated to receive on average a total of 42.9 bppn, whereas users of nets received a total of 11.2 bppn (Table 3), suggesting that about one third of mosquito bites could not be prevented solely by using a net. This high level of residual biting is due to high levels of biting outdoors, as well as indoor biting before going to bed. In this setting, peak biting intensity was between 2 and 4 am when most people were in bed. If biting peaks were earlier, when residents are still outdoors, or indoors before going to bed, residual biting would be even higher. Entomological studies [18] have shown that indoor biting continues well into the day. In this study, no data were available for daytime HLC rates. True residual biting exposure is therefore likely to be higher than what was estimated, since there is generally no protection against daytime biting.

If vector control functions optimally, then the killing of mosquitoes through contact with the insecticide on the net when seeking a blood meal would reduce the mosquito population sufficiently to provide protection for non-users of nets as well, reducing overall biting exposure. Given the very high level of net use in this study, it is possible that mosquito biting would be even more intense if nets were not used or used less consistently. On the other hand, it is possible that pyrethroid resistance made the nets less effective at killing mosquitoes [11, 12]. Overall, biting (and hence residual biting) could be reduced by using dual-active-ingredient (AI) nets, such as chlorfenapyr-pyrethroid nets in place of the standard pyrethroid nets used during this study, or by IRS with non-pyrethroid insecticides. However, randomised trials comparing dual-AI nets against standard pyrethroid nets [19, 20] showed that participants in the dual-AI study arm, whilst having lower prevalence of infection and lower incidence of malaria, still suffer considerable levels of malaria disease burden, consistent with high levels of residual transmission.

Previous studies in Kenya, Mozambique, Equatorial Guinea and Zambia [21–24] combined data on mosquito biting intensities and human behaviour to evaluate residual biting in different settings due to a variety of vector species. In these studies, most residual biting was estimated to be due to indoor biting exposure, in contrast to the present study, where most residual biting occurred outdoors. These differences in the nature of residual biting are due to differences in times of peak biting and the amount of time people spend indoors before going to bed. In this study, people spent on average about 1 h indoors before going to bed, thus limiting their residual indoor biting exposure. Based on mosquito feeding behaviour studies from across Africa and a small number of human behaviour studies, a systematic review [25] estimated that on average 79% of bites occurred during the hours that people are in bed, and hence preventable by using bednets. The study concluded that due to increased exposure to outdoor biting, residual malaria transmission is steadily increasing, noting the dearth of human behaviour data to assess indoor exposure to biting. For malaria control programmes, to determine what additional interventions to deploy in the future to reduce exposure to vector mosquito bites, it is important to know whether residual biting is due primarily to indoor biting or outdoor biting. We would therefore recommend that at least small-scale studies on residual biting exposure be conducted to inform evidence-based policy decisions on vector control interventions. A better understanding of peri-domestic activities of people before they retire indoors and indoor activities they do before sleeping could help to better target communications and complementary interventions to reduce residual biting.

High heterogeneity was found in average nightly HLCs experienced by collectors between communities, ranging from 4 to 125 bites per night for outdoor collectors, and from 5 to 104 bites per night for indoor collectors (Supplementary material, Table S2). Most of this variation is due to distance from the river, which was strongly inversely associated with biting exposure, even after adjusting for altitude (Table 4). Age was a risk factor for biting due to differences among age groups in time of going indoors and going to bed, with young children receiving the lowest number of bites. Going indoors later was associated with biting, since outdoor HBR was higher than indoor HBR from about 10 pm (Fig. 1). Bednet use was necessarily associated with reduced biting exposure, since in assigning bites to study participants it was assumed that once under a bednet, a person received no further bites. Screening at the windows was also protective against biting but was rare in this study population (about 4% of respondents). The lack of association between socio-economic status and biting should be seen as a limitation of the data rather than as a true lack of an association, since bites were assigned to people without being able to take account of the quality of the dwelling they occupied. However, in the rural settings of this study, the population was socio-economically fairly homogeneous, with little variation in housing structure.

Similar to many other studies, children were at higher risk of malaria infection than adults (Table 5), despite their lower exposure to bites. Lower infection prevalence in adults, despite high biting exposure, may be a result of their partial immunity in a setting of high exposure to malaria parasites. Counter-intuitively, we found only very partial evidence of an association between exposure to bites and malaria infection. The sample available for this part of our analysis was relatively small, resulting in low power to detect an association.

A strength of the study is that it measured mosquito biting and concomitant human behaviours in 30 separate communities close to and distant from the Niger river, and at a range of altitudes. Households selected for HBR measurements and individuals selected for human behaviour interviews were randomly selected and hence representative of the communities in which they were located. The number of sampling points for entomological collections was larger than those in previous studies, and the sample of individuals contributing to human behaviour data also exceeded those in similar studies in the past.

Since this study did not include data on sporozoite infection, we cannot definitively conclude that malaria transmission was higher outdoors than indoors, despite substantially higher exposure to *Anopheles* bites outdoors than indoors. However, previous studies in southern Mali found no significant difference in sporozoite infection prevalence between indoor- and outdoor-caught mosquitoes [26]. A further shortcoming of our study was that mosquitoes were only identified to species complex (*An. gambiae* s.l.). Sibling species within the *An. gambiae* complex exhibit distinct biting behaviours, and may not be present in equal proportions indoors and outdoors. Keita et al. [26] found that *An. coluzzii* was the overwhelmingly predominant species in southern Mali [26]. We caution that any extrapolation of our finding of higher outdoor biting exposure to higher outdoor malaria transmission is at best tenuous.

A further limitation of the study was that human behaviour data were self-reported and collected cross-sectionally only. Bednet use may have been seen by respondents as a socially desirable behaviour, and thus over-reported. Further, no behavioural data on time of getting up and leaving the house were collected, and no entomology daytime biting data were available. The assumptions of zero indoor biting exposure whilst sleeping under a bednet or whilst indoors in the morning after getting up and no outdoor biting exposure after leaving the house will have resulted in an underestimate of residual biting. As already stated, a further limitation is that residual biting was based on the personal protection that is provided by bednets, ignoring the reductions in biting that are due to the community effect resulting from mosquitoes being killed by the insecticide on the net. This would result in an overestimate of the proportion of biting that is “residual”. However, this study clearly shows that even if using a net, the number of bites that people in this setting are exposed to is very high, and further interventions are needed to reduce exposure to mosquito bites, particularly whilst outdoors.

## Conclusions

This study demonstrates that sleeping under a net will substantially reduce exposure to vector mosquito bites, but the protection they offer is only partial. ITNs will remain the cornerstone of malaria vector control for the foreseeable future, but in settings like the Koulikoro region with very high vector mosquito biting intensity despite high net usage, people remain exposed to high levels of biting, and hence of malaria, particularly outdoors. Effective supplementary interventions will be required to reduce the very high malaria burden in countries like Mali.

## Supplementary Information

Supplementary material 1.

Supplementary material 2.

Supplementary material 3.

Supplementary material 4.

## Data Availability

De-identified data are available from the corresponding author on reasonable request. Following publication of forthcoming secondary analyses of trial data, the de-identified trial dataset will be posted on a data repository for long-term curation.
