# Supplementary figures and images for: High exposure to malaria vector bites despite high use of bednets in a setting of seasonal malaria in southwestern Mali: the urgent need for outdoor vector control strategies
Source: Parasit Vectors. 2025 Jul 9;18:274. doi: 10.1186/s13071-025-06818-8 (PMC12243244; doi:10.1186/s13071-025-06818-8)

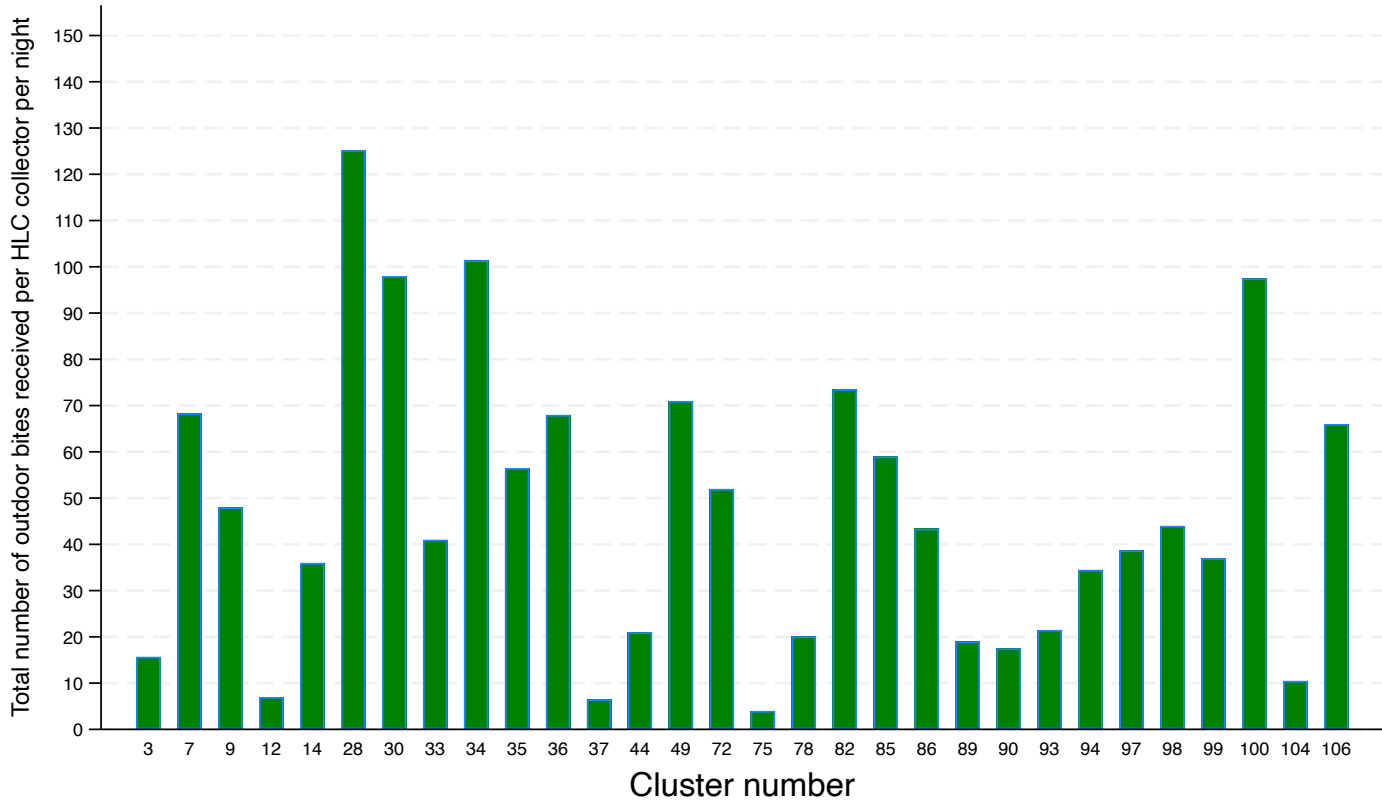

Supplement: Supplementary file 1 — Supplementary material 1. [file 13071_2025_6818_MOESM1_ESM.pdf]

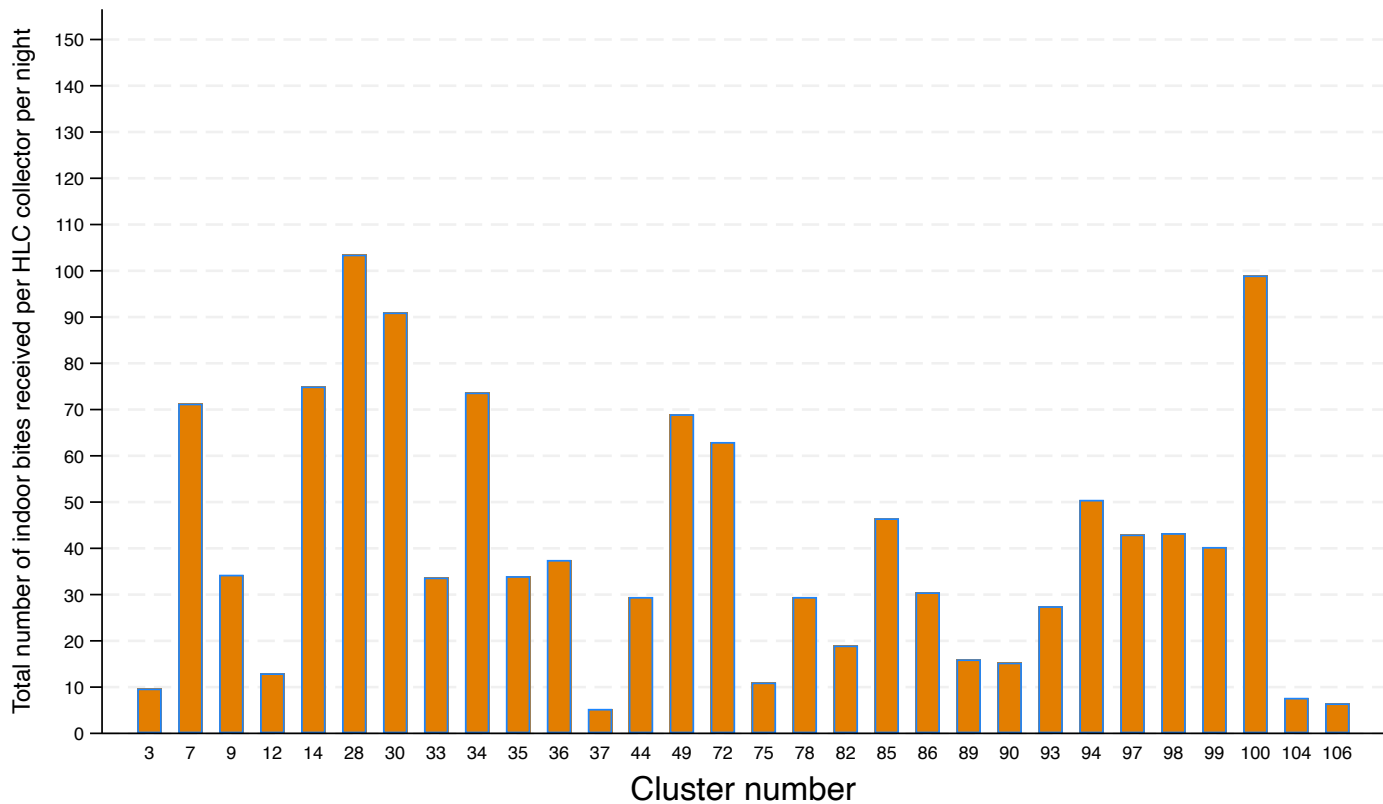

Supplement: Supplementary file 2 — Supplementary material 2. [file 13071_2025_6818_MOESM2_ESM.pdf]

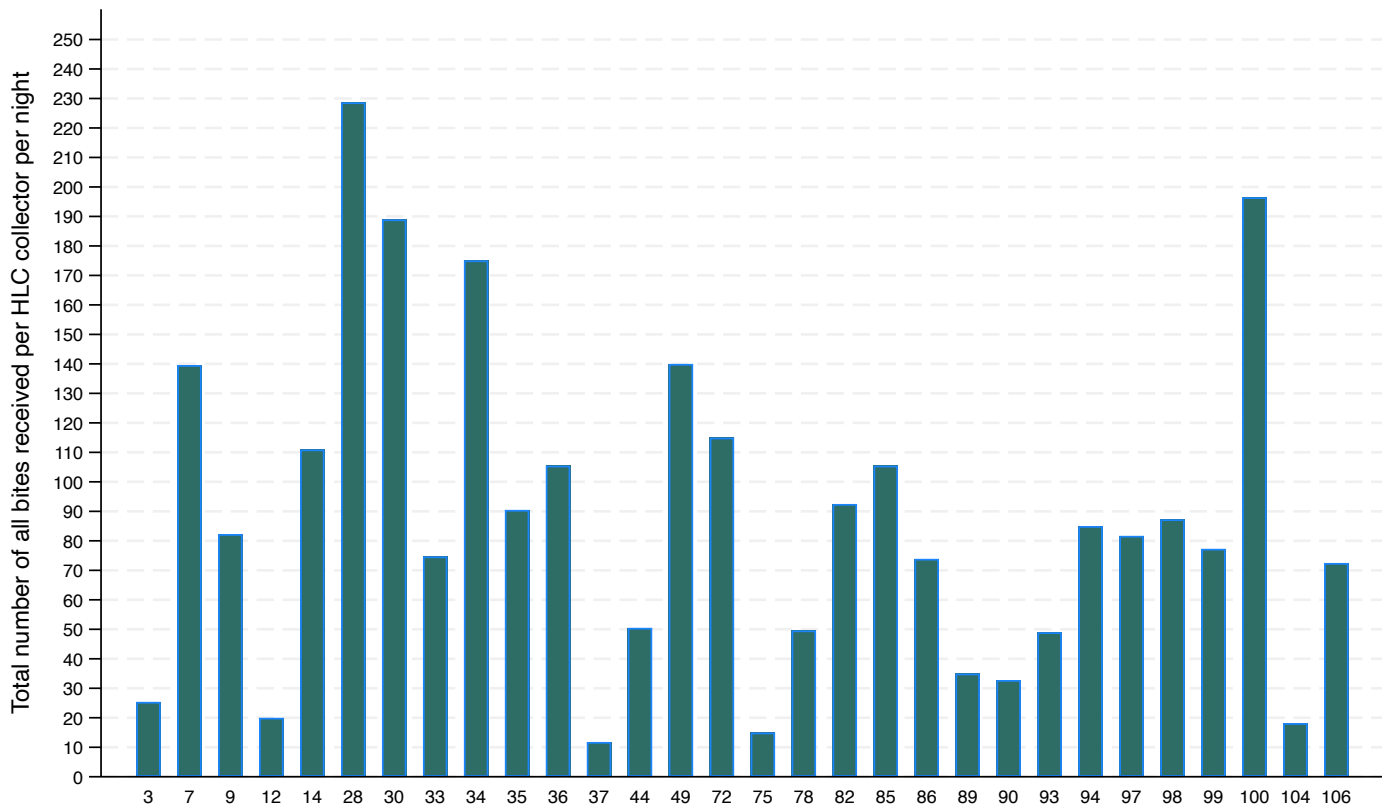

Supplement: Supplementary file 3 — Supplementary material 3. [file 13071_2025_6818_MOESM3_ESM.pdf]
