# Supplementary material for: High exposure to malaria vector bites despite high use of bednets in a setting of seasonal malaria in southwestern Mali: the urgent need for outdoor vector control strategies
Source: Parasit Vectors. 2025 Jul 9;18:274. doi: 10.1186/s13071-025-06818-8 (PMC12243244; doi:10.1186/s13071-025-06818-8)
Supplement: Supplementary file 4 — Supplementary material 4. [file 13071_2025_6818_MOESM4_ESM.docx]

Supplementary material, Table S1. Distribution of socio-demographic characteristics and bed net use between individuals included in the analysis and individuals excluded due to data missingness (preventing estimation of individual exposure to biting)

|  | | Total sample (N = 2,997) | | | Individuals with missing biting exposure estimates (N = 2,754) | |
| --- | --- | --- | --- | --- | --- | --- |
|  | n | | **Distribution (%)** | n | | **Distribution (%)** |
| Household wealth quintiles |  | |  |  | |  |
| Poorest | 511 | | **17.1** | 465 | | **16.9** |
| Poorer | 516 | | **17.2** | 564 | | **20.5** |
| Middle | 602 | | **20.1** | 670 | | **24.3** |
| Richer | 657 | | **21.9** | 498 | | **18.1** |
| Richest | 711 | | **23.7** | 557 | | **20.2** |
| Total | 2,997 | | **100.0** | 2,754 | | **100.0** |
| Household distance to the river (km) |  | |  |  | |  |
| <5 | 279 | | **9.3** | 253 | | **9.2** |
| [5-10[ | 932 | | **31.1** | 681 | | **24.7** |
| [10-60[ | 1,786 | | **59.6** | 1,820 | | **66.1** |
| Total | 2,997 | | **100.0** | 2,754 | | **100.0** |
| Mean |  | | **21.2** |  | | **23.3** |
| Household altitude (meters) |  | |  |  | |  |
| ]320-380] | 912 | | **30.4** | 611 | | **22.2** |
| ]380-400] | 1,386 | | **46.3** | 1,344 | | **48.8** |
| ]400-510] | 699 | | **23.3** | 799 | | **29.0** |
| Total | 2,997 | | **100.0** | 2,754 | | **100.0** |
| Mean |  | | **397.0** |  | | **397.4** |
| Age group (years) |  | |  |  | |  |
| 0-4 | 390 | | **13.0** | 385 | | **14.0** |
| 5-14 | 1,157 | | **38.6** | 1,041 | | **37.8** |
| 15-44 | 959 | | **32.0** | 885 | | **32.2** |
| 45-95 | 490 | | **16.4** | 441 | | **16.0** |
| Total | 2,996 | | **100.0** | 2,752 | | **100.0** |
| Mean |  | | **22.5** |  | | **22.2** |
| Sex |  | |  |  | |  |
| Male | 1,488 | | **49.7** | 1,323 | | **48.0** |
| Female | 1,509 | | **50.4** | 1,431 | | **52.0** |
| Total | 2,997 | | **100.0** | 2,754 | | **100.0** |
| School level (highest grade completed) |  | |  |  | |  |
| Never attended school | 1,838 | | **62.4** | 1,832 | | **67.6** |
| Primary | 910 | | **30.9** | 736 | | **27.2** |
| Secondary/Higher | 199 | | **6.8** | 142 | | **5.2** |
| Total | 2,947 | | **100.0** | 2,710 | | **100.0** |
| Bed net use last night |  | |  |  | |  |
| No | 205 | | **6.8** | 264 | | **9.6** |
| Yes | 2,792 | | **93.2** | 2,490 | | **90.4** |
| Total | 2,997 | | **100.0** | 2,754 | | **100.0** |

Supplementary material, Table S2. Between cluster variation in the average total number of bites received per HLC collectors per night (from 6pm to 6am) (N = 30 entomological clusters)

| Total number of bites received per HLC collectors per night | Mean | SD | Median | Min | Max |
| --- | --- | --- | --- | --- | --- |
| Outdoors | 46.7 | 31.4 | 42.2 | 4.0 | 125.2 |
| Indoors | 41.0 | 28.2 | 34.2 | 5.3 | 103.5 |
| Total | 87.7 | 56.4 | 82.0 | 11.8 | 228.7 |

Supplementary material, Figure S1. Between cluster variation in the average total number of outdoor bites received per HLC collectors per night (from 6pm to 6am) (N = 30 entomological clusters)

Supplementary material, Figure S2. Between cluster variation in the average total number of indoor bites received per HLC collectors per night (from 6pm to 6am) (N = 30 entomological clusters)

Supplementary material, Figure S3. Between cluster variation in the average total number of all bites received per HLC collectors per night (from 6pm to 6am) (N = 30 entomological clusters)
